# Supplementary material for: Perirhinal firing patterns are sustained across large spatial segments of the task environment
Source: Nat Commun. 2017 May 26;8:15602. doi: 10.1038/ncomms15602 (PMC5458559; doi:10.1038/ncomms15602)
Supplement: Supplementary Information — Supplementary Figures, Supplementary Tables and Supplementary References [file ncomms15602-s1.pdf]

Supplementary Figure 1

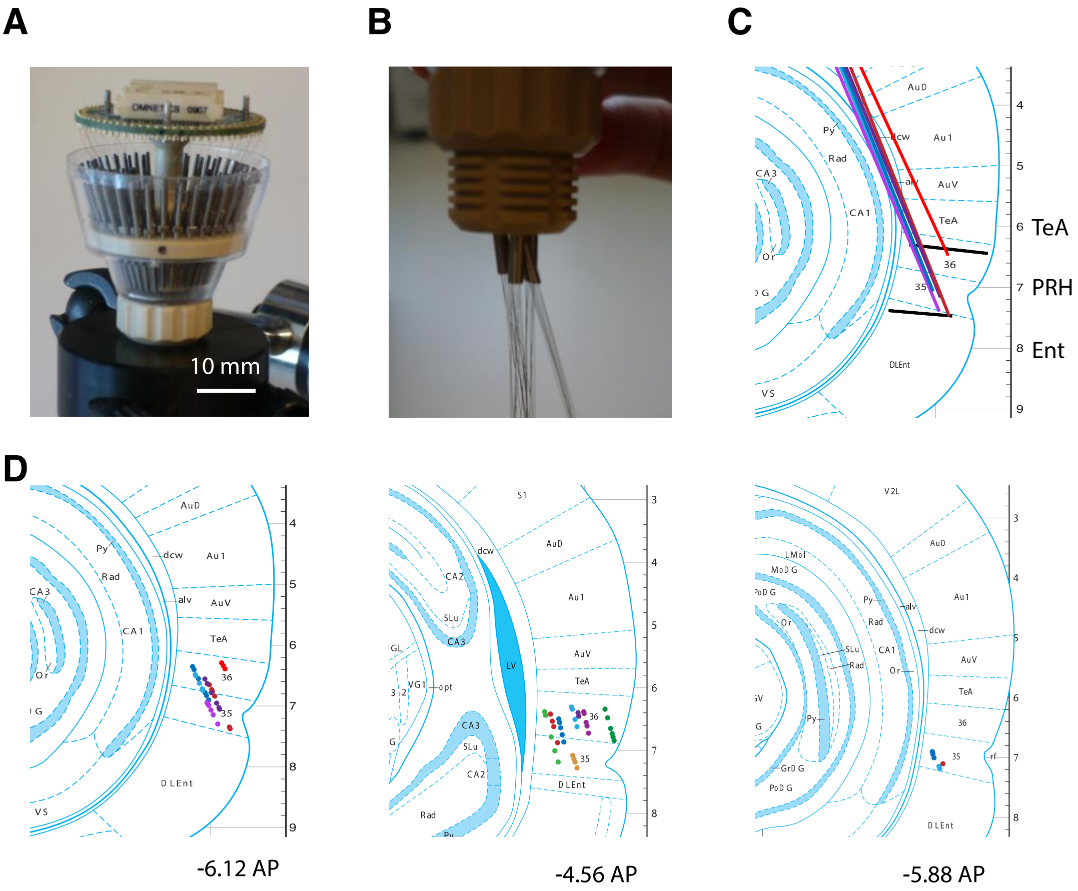

Supplementary Figure 1. Recording technology and perirhinal recording locations. (A) Side view of the 144-channel microdrive (quad drive) with four bundles, each of which holds 9 tetrodes (8 for recording, 1 reference). Lead screws and connector board are visible; the drive is placed in a holder, occluding view on bottom part where tetrodes exit the drive. (B) Side view of bottom part of the microdrive, showing how the four bundles (consisting of tetrodes that have not been cut yet to desired lengths) emerge from the drive at different angles. (C) Tetrode tracks targeting the perirhinal cortex (areas 35 and 36) in an example rat. Each color represents a different tetrode track, overlaid on a section from Paxinos and Watson[1] Near-horizontal, black lines delineate the boundaries between perirhinal cortex (PRH), temporal association area (TeA) and entorhinal cortex (Ent; scale in mm). (D) Overview of final tetrode recording sites (endpoints) in PRH of the rats analyzed, projected onto three anterior-posterior levels (-6.12 AP, -4.56 AP and -5.88 AP). Each color represents a distinct tetrode track. Only recordings made from area 35 and 36 are shown here.

Supplementary Figure 2

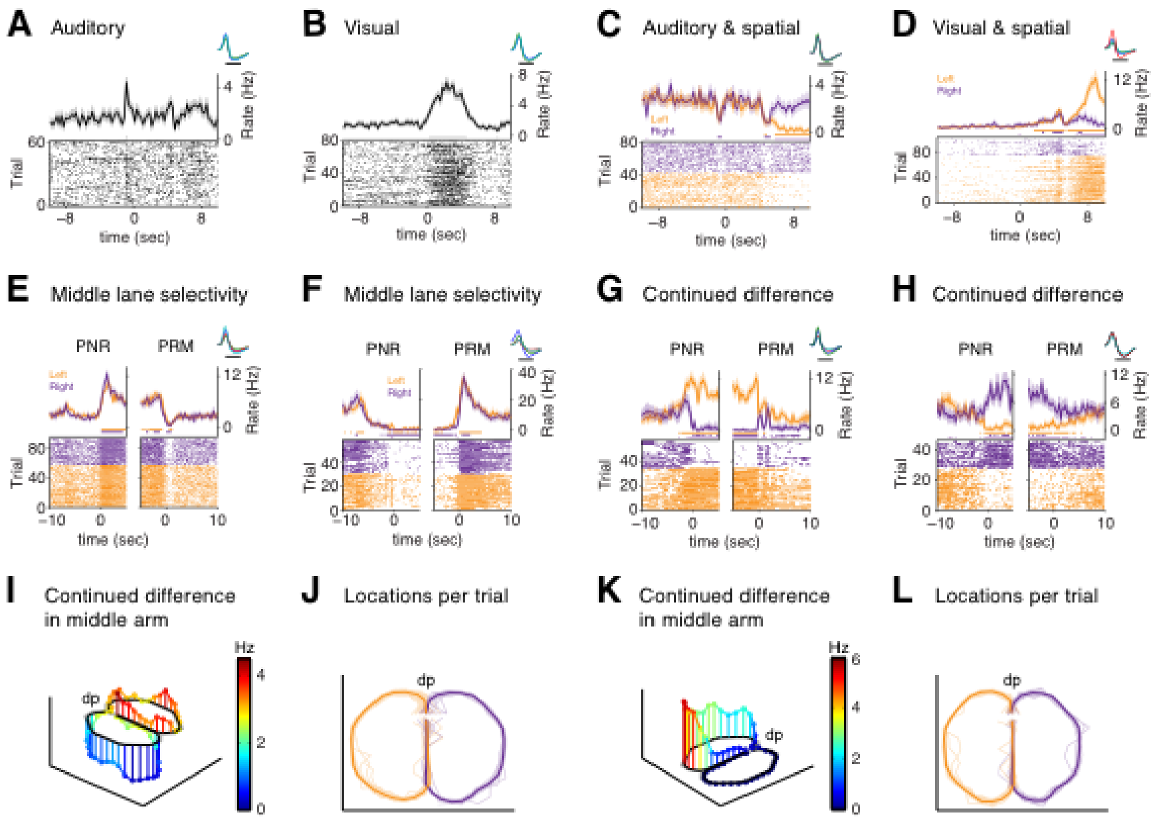

Supplementary Figure 2. Perirhinal neurons showing singular responses to sensory events or more complex response profiles, including retrospective coding. All panels represent peri- stimulus time histograms of different individual cells, illustrating a diversity of perirhinal coding capacities beyond the main profile showing sustained discriminatory firing described in the main text. (A) Neuron showing a transient firing-rate increase (shown as mean ± s.e.m) upon the auditory cue (onset at t = -1.0 s) which indicates that the rat can initiate a new trial. Top inset: superimposed spike waveforms from four tetrode leads (scale bar: 0.5 ms). (B) Neuron showing a waxing-waning response to the visual cue (onset at t=0, offset when the rat passed the Point of No Return at about t=5 s). Note that the firing-rate increment of this cell is terminated by the time the animal arrives at the decision point. Grey horizontal lines below average rate curves in (A,B) indicate significant differences from baseline (Wilcoxon signed rank test, $\alpha = 0.05$). (C) Neuron showing a complex response profile, consisting of a transient decrease in firing rate upon sound cue onset (at t = -1.0 s), followed by a differential response evolving when the rat enters the left (orange) versus right (purple) arm at about t = 4.5 s. (D) Neuron showing a complex response profile, consisting of a progressively incremental firing rate initiated on visual cue onset (“ramping” behavior), followed by a discriminatory response when the rat enters the left (orange) versus right (purple) arm at about t = 5 s. Although the cells in (C, D) may superficially resemble CA1 splitter cells showing prospective coding of place, it should be noted that the traces for Left and Right trials only start diverging at around t = 4.5 to 5.0 seconds, when the rat passes the spatial decision point. (E) Neuron showing a selective firing-rate decrease when the rat was positioned in the middle lane of the figure-8 maze (color code as above; t=0 in left and right graph is passage of PNR and PRM, respectively; both Left and Right arm trials show a sustained enhancement with respect to the middle lane). (F) As in (E), but now for a neuron showing a selective firing-rate increase for the middle lane. (C-F) Orange and purple horizontal bars below average rate curves indicate statistical significance (Wilcoxon signed rank test, $\alpha = 0.05$) of trials to Left and trials to Right with respect to the baseline (intertrial interval), respectively. Lilac horizontal bars mark significant differences between Left (orange) and Right (purple) trials (Mann-Whitney U test with FDR correction, $\alpha = 0.05$). (G, H) Two example neurons showing a continuation of Left-Right discriminatory firing, after the rat had returned to the middle lane, for several seconds. The neuron in (G) maintained a higher firing rate when coming from the Left as compared to the Right arm. The neuron in (H) showed roughly the opposite effect, having a higher firing after passing the PRM when coming from the Right vs. Left side. (I) Example cell showing a similar middle-arm retention effect in a spatial plot of firing rate (color-coded along the maze trajectories; side view). Enhanced firing observed for the Right arm is maintained when the rat travelled through the middle lane until reaching the front blocking wall. (J) Behavioral trajectories of the rat from which the unit in (I) was recorded (thick line: mean trajectory across the session; thin lines: single-trial runs; top view). Whereas the average paths slightly differed when the rat returned to the initial segment of the middle lane, coming from the Left versus Right arm, the paths overlapped in the middle-lane segment visited subsequently. (K, L) Second example cell showing a retention effect in spatial plot of firing rate (K; behavioral trajectories: L). Cells in (I, K) are different from those in (G, H). DP, decision point.

Supplementary Figure 3

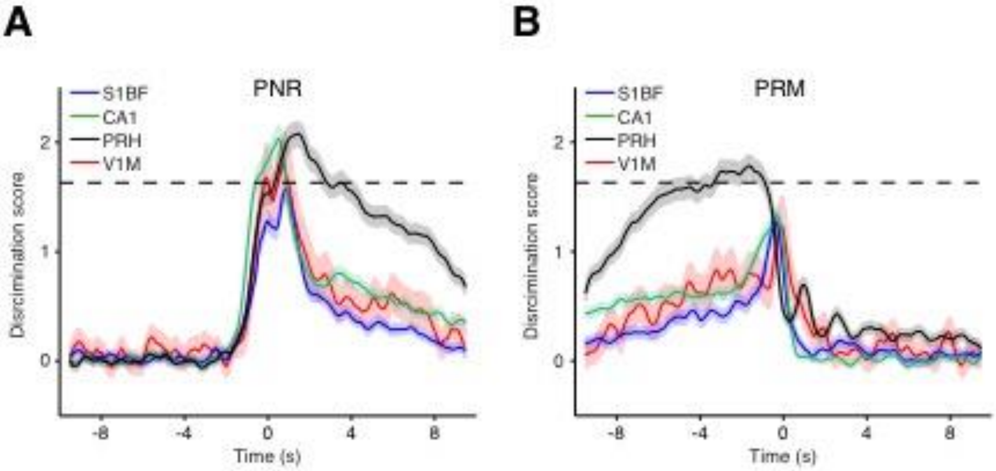

Supplementary Figure 3. Time course of spatial Discrimination Score for CA1, perirhinal, somatosensory and visual cortical populations. (A) Average Discrimination Score (mean ± s.e.m. across cells) plotted as a function of time relative to crossing the point of no return (PNR). (B) Idem as (A) but now relative to crossing the point of returning to the middle lane (PRM). Threshold for significance (one tailed Z-statistic, $\alpha < 0.05$) lies at 1.63 (marked by dashed line). The average duration of the time segments showing a significant Left-Right Discrimination Score was 1,96 ± 0.10 s (S1BF), 2,43 ± 0.11 s (CA1), 4,11 ± 0.17 s (PRH) and 2,51 ± 0.32 s (V1M; these durations cannot be estimated from the graphs because they are based on individual trial segments). The duration for PRH cells was significantly higher than for the other three areas (Kruskal-Wallis and post-hoc Tukey-Kramer tests with a 95% confidence interval). The average time it took rats to pass from the PNR to PRM points was 8.83 ± 0.36 s. That Discrimination Scores were elevated for shorter time periods than these behavioral run periods is explained by the nature of time-locked representations and given the behavioral variability in the speed it takes a rat to complete a trial. In other words, because some trials are completed rapidly (within a few seconds) and others take longer to complete, a behavioral scattering or jitter occurs relative to t=0 s, causing a dilution effect and thus a decline of the Discrimination score in (A) and a slow rise in (B). Furthermore, the Discrimination score is computed as the T- statistic involving the standard deviation (see Methods), clarifying that this measure will decrease when the standard deviation of the distribution of shuffled firing-rate differences increases, which occurs because of behavioral variability. It should be noted that the curves for PRH, CA1 and S1BF are based on large cell samples (518, 660, and 458, respectively) whereas the curve for visual cortex (V1M) is based on a relatively small sample (43 cells). Overall, this figure illustrates that PRH cells show significantly more sustained firing than cells in the other three areas, but also that it is more appropriate to quantify these firing patterns as a function of space rather than time.

Supplementary Figure 4

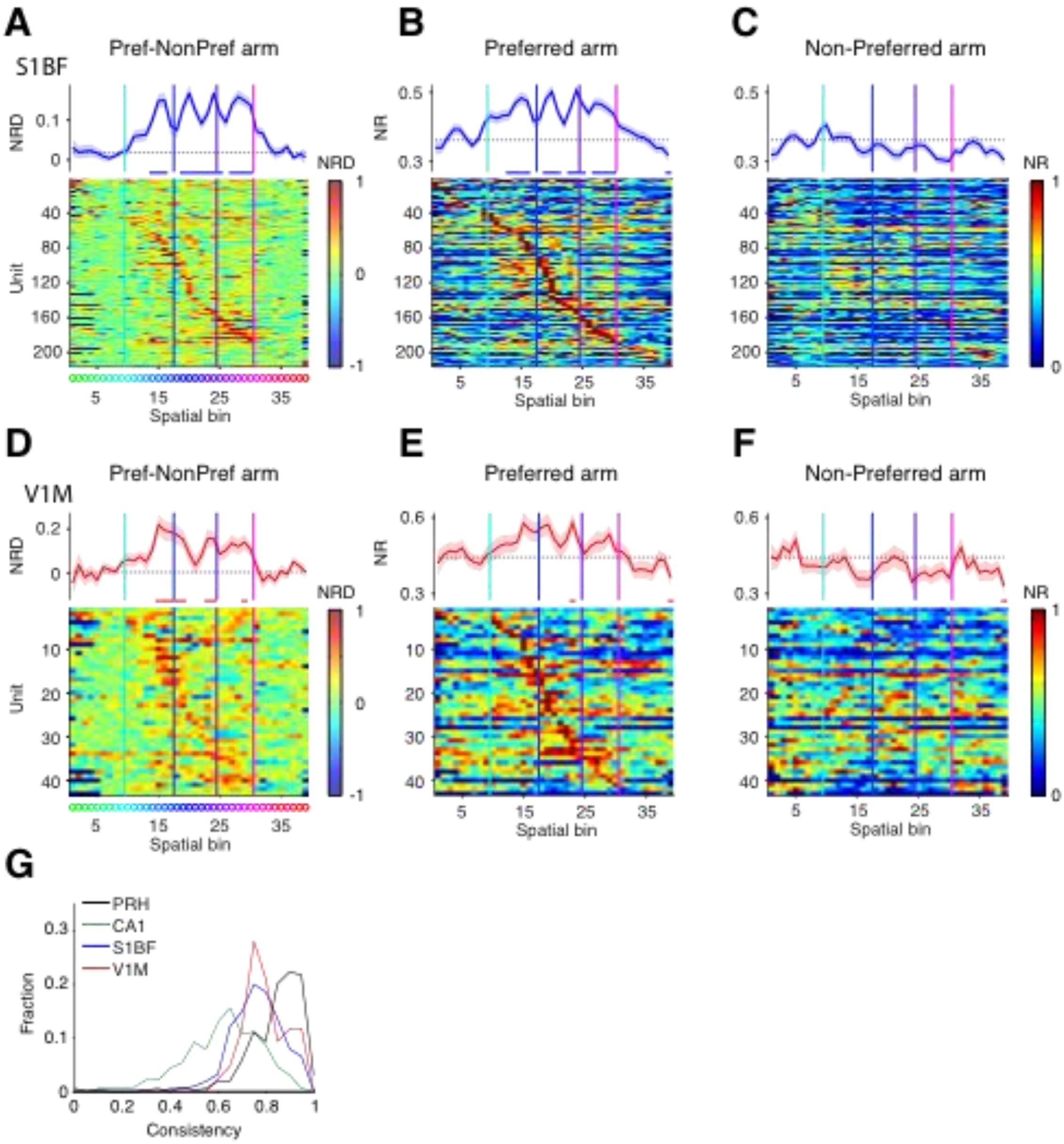

Supplementary Figure 4. Firing patterns of neurons in somatosensory and visual cortex as a function of place. This figure is complementary to Fig. 4 in which perirhinal and hippocampal patterns are shown. (A-C) Population of 216 neurons in somatosensory cortex. Vertical colored lines correspond to the spatial decision point (cyan), PNR (blue), reward site (purple) and PRM (pink; see Fig. 3 for other plot details). (D-F) Population of 43 neurons in primary visual cortex (monocular field, V1M). In (A) and (D), the Normalized Rate Difference (NRD) is plotted as a function of linearized maze position. In (B) and (E), the spatial profile is shown for the preferred arm of each single unit. Cells have been arranged from top to bottom according to the spatial bin where the cell reached its peak firing rate. Color code pertains to normalized firing rate (NR). (C) and (F) Same as (B, E) but now for the non-preferred arm of each cell. (G) Frequency histogram (as fraction of cells with discrimination scores > 0.5) of the Consistency of spatial Left-Right differences in firing activity for S1BF (N = 216), area CA1 (N = 373), PRH (N = 349) and V1M (N = 43). The PRH population showed a significantly higher consistency than the other three areas (Wilcoxon two-sample rank test; S1BF-PRH, Z = -8.98, P = 0; CA1-PRH, Z = 17.76, P = 0; V1M-PRH, Z = -3.26, P = 0.001).

Supplementary Figure 5

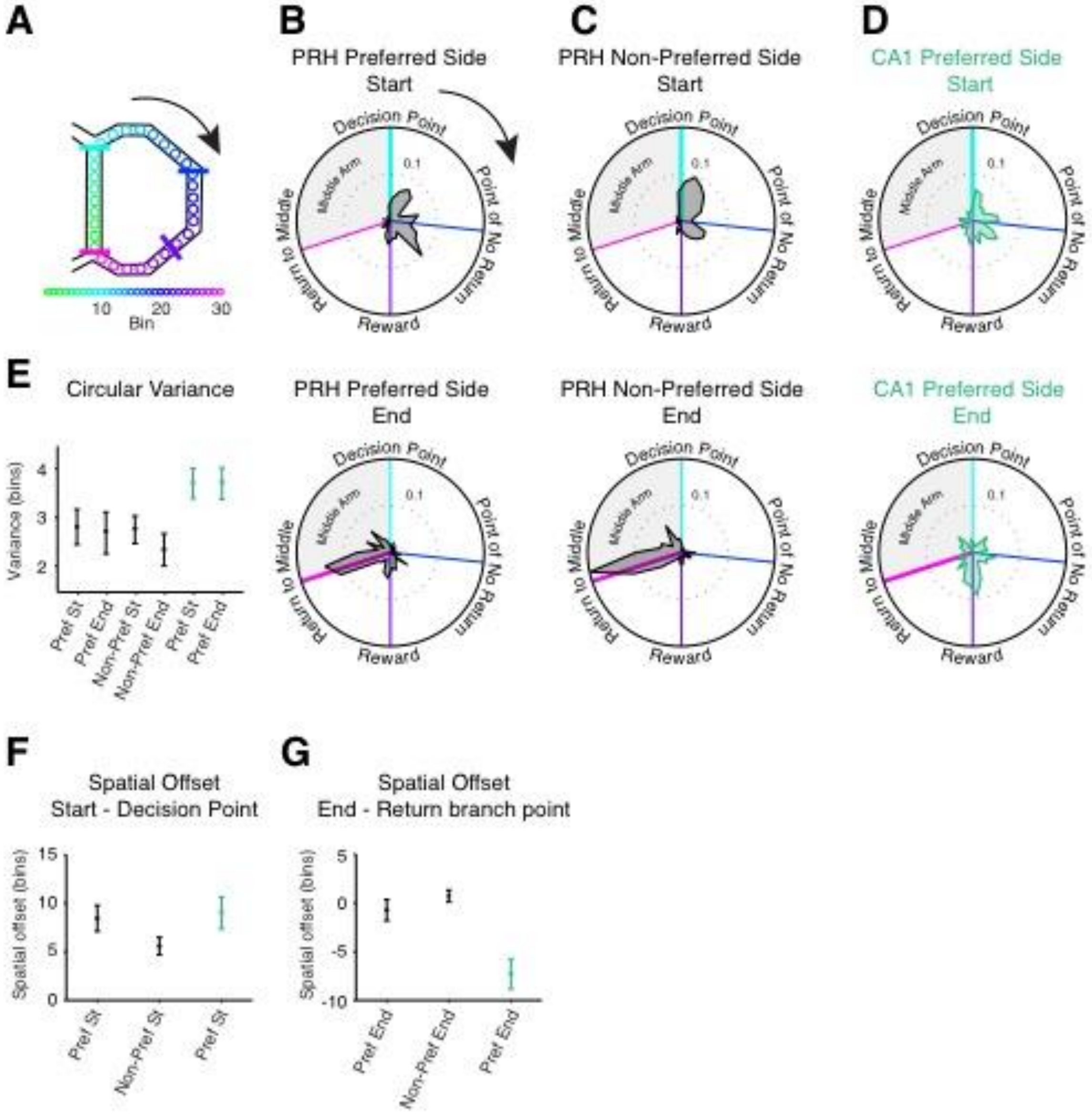

Supplementary Figure 5. Start and end points of perirhinal and hippocampal firing fields relative to task-relevant points on the figure-8 maze. (A) Color code of the distributions of bins on the maze. To avoid artifacts due to edge effects, the route travelled on a full trial was taken to be circular, with bin 1 (start of middle lane, coinciding with the return branch point; green) being contiguous with bin 30 (end of the side arms; magenta). Runs on the left and right side arms are included in the same projection. (B) Distribution of start points (top) and end points (bottom) of PRH firing fields on their preferred arm along the running trajectory are rendered in polar plots, relative to task-relevant points on the maze (decision point, point of no return, reward site and return branch point). For each cell, start and end points were determined by subtracting the mean firing rate across both sides (i.e., the baseline) from the preferred-side firing rate (see Methods). Start and end points are defined according to locomotion direction during task performance. Fraction of occurrence is plotted along the radial axis. (C) Distributions of start (top) and end points (bottom) of firing fields of PRH units for the non-preferred side arm (see Methods). (D) Distribution of start points (top) and end points (bottom) of CA1 units for the preferred side. PRH start points for the non-preferred side were mostly concentrated at spatial locations reached by the rat shortly after passing the decision point, while start points for the preferred side were more distributed. A subset of PRH units showed start points between the point of no return and reward site of the preferred arm. The distribution of CA1 start points was more spread out than those of PRH cells for their non- preferred side. PRH end points for both the preferred and non-preferred arm were strongly co- localized with the return branch point (pink line). (E) Circular variance and bootstrapped 95% confidence intervals of start and end points expressed in maze bins. Black symbols refer to PRH, green to CA1 (St, start point). The variance of both start and end points of PRH firing fields was smaller than that of CA1 place fields, a finding that applied to both PRH firing-rate changes on the preferred and non-preferred arm (95% confidence interval, based on bootstrapping, for all four comparisons: Pref St CA1 vs. Pref St PRH; Pref St CA1 vs. Non-Pref St PRH; Pref End CA1 vs. Pref End PRH; Pref End CA1 vs. Non-Pref End PRH; See Supplementary Table S3). (F) Mean and 95% confidence intervals of the spatial offset between the start points of firing fields and the decision point (Bin 9). (G) Mean and 95% confidence intervals of offsets of end points with respect to return branch point (bin 1). The mean and confidence intervals are expressed in spatial bins. Negative values are assigned to positions reached by the animal before the reference point (decision point or return branch point) and positive values for positions reached thereafter. The spatial offsets of start and end points of PRH firing fields were significantly different from those of CA1 cells for 3 out of 4 comparisons (95% confidence interval, based on bootstrapping, for Pref CA1start-DP vs. Non-Pref PRHstart-DP; Pref CA1end-RBP vs. Pref PRHend-RBP; Pref CA1end-RBP vs. Non-Pref PRHend-RBP; n.s. for Pref CA1start-DP vs. Pref PRHstart-DP; DP, decision point; RBP, return branch point).

Supplementary Tables

Supplementary Table 1: Cells discriminating left versus right arms of the figure-8 maze were found in all rats and main areas of interest. Numbers shown below are percentages of cells showing significant Left-Right discrimination ($\alpha = 0.05$, Wilcoxon signed-rank test), split up per rat (rows) and brain region (columns). In between brackets, fractions show absolute counts of significantly modulated cells divided by all cells recorded from a given area and rat.

Rat index	PRH (N=374/518)	CA1 (N=404/660)	S1BF (N=182/458)	Total number of cells per rat
1	75.7 % (N=184/243)	62.6 % (N=261/417)	40.4 % (N=113/280)	N=940
2	70.6 % (N=185/262)	62.7 % (N=126/201)	35.7 % (N=25/70)	N=533
3	38.5 % (N=5/13)	40.5 % (N=17/42)	40.7 % (N=44/108)	N=163

Supplementary Table 2: Overview of perirhinal neuron fractions showing different types of firing rate modulation on the figure-8 maze. The total number of neurons considered was the group of 374 (out of 518) PRH neurons showing significant modulation. 'Selective for Left arm' refers to a significantly different rate between the Left and Middle arms, and implies that the Right arm and Middle arm responses were not significantly different. 'Selective for both Left and Right arm' implies that both arm responses were significantly different from the middle arm, but also the contrast Left vs. Right was significantly different (e.g. firing rate increases in left arm increase, but decreases in right arm; therefore, this type of modulation was not partitioned into increments or decrements). Percentages of CA1, S1BF and V1M showing significant modulations did not radically diverge from the proportions shown for PRH, but are not presented here because they were locally restricted, for instance due to place-field responses in area CA1.

Percentage of PRH neurons	Selective for Left arm	Selective for Right arm	Selective for Middle arm	Selective for both Left and Right arm
Total percentage modulated	39.6%	16.9%	10.9%	32.6%
Total percentage showing increase	17.1%	13.4%	8.0%	-
Total percentage showing decrease	22.5%	3.5%	2.9%	-

Supplementary Table 3: Variance of start and end points of firing fields across the maze and their spatial offsets relative to the decision point and return branch point, respectively. The mean variance, spatial offsets and confidence intervals are expressed in bins. One full lap on the maze consists of 30 bins. Negative values are assigned to positions reached by the animal before the reference point (decision point or return to middle) and positive values for positions reached thereafter. CI-low, lower boundary of the 95% confidence interval. CI-high, higher boundary of the 95% confidence interval.

	Preferred		Preferred		Non-Preferred	
Area	CA1 Start	CA1 End	PRH Start	PRH End	PRH Start	PRH End
**Variance**	3.72	3.73	2.81	2.72	2.77	2.34
CI-low	3.39	3.38	2.45	2.26	2.47	2.01
CI-high	4.01	4.03	3.17	3.11	3.03	2.67
**Spatial offset**	9.09	-7.27	8.41	-0.68	5.54	0.70
CI-low	7.36	-8.79	7.10	-1.36	4.68	0.11
CI-high	10.64	-5.79	9.78	0.36	6.49	1.32

Supplementary References

1 Paxinos, G. & Watson, C. *The rat brain in stereotaxic coordinates*. (Academic press, 2007).
